# CTA Study of Ruptured Aneurysms of the Posterior Communicating Artery

**DOI:** 10.1155/2022/5774735

**Published:** 2022-09-14

**Authors:** Zibo Zhou, Jinlu Yu

**Affiliations:** Department of Neurosurgery, First Hospital of Jilin University, Changchun 130021, China

## Abstract

**Background:**

Only a few reported studies have used computed tomography angiography (CTA) to image ruptured aneurysms at the junction of the internal carotid artery (ICA) and posterior communicating artery (PcomA) in the context of the adjacent arteries. Therefore, we performed such a study using a GE Workstation.

**Methods:**

The parameters of each aneurysm and its adjacent arteries were measured. Then, statistical assessments were performed to compare the parameters of the aneurysm side and the lesion-free (control) side.

**Results:**

Sixty-three patients were included in this study. The average age was 62.1 ± 11.0 years, and the ratio of males to females was 0.8 : 1. The measurement results showed that the mean aneurysmal height was 5.2 ± 2.3 mm, the mean width was 4.7 ± 2.2 mm, and the mean neck width was 4.5 ± 1.9 mm. On the aneurysm side, the intradural ICA diameter was 4.34 ± 0.90 mm, and the diameter of the ICA at its termination was 3.55 ± 0.72 mm. A fetal-type PcomA was found in 52.4% of aneurysms. The other measured parameters were also provided. Statistical results showed that the height of the aneurysm was larger than the width (*P* < 0.05). The intradural ICA diameter, the ICA diameter at termination, the intradural ICA length, and the angle between the ICA and PcomA were larger in the aneurysm group than in the control group (*P* < 0.05).

**Conclusions:**

This CTA study showed that the ruptured PcomA aneurysm was often wide-necked, nonspherical, and approximately 5 mm in size. In the presence of a ruptured PcomA aneurysm, the affected intradural ICA became thicker and longer than the contralateral control ICA, and the aneurysm significantly reduced the angle between the ICA and the PcomA.

## 1. Introduction

In general, the junction of the internal carotid artery (ICA) and the posterior communicating artery (PcomA) is a common location for the formation of intracranial aneurysms, accounting for approximately 15-25% of all intracranial aneurysms and 50% of all ICA aneurysms. [1] Clinically, the aneurysm at this location is commonly called a PcomA aneurysm. Because of the anatomical variations in PcomA aneurysms and their adjacent arteries, some aneurysms are among the easiest to treat either surgically or endovascularly, while others are others are among the most difficult [1, 2]. Therefore, the parameters of the individual PcomA aneurysm and its adjacent arteries are very important for the treatment [3–5].

Previous studies on PcomA aneurysms and their adjacent arteries have mostly been based on catheter angiography or anatomical specimens [3, 4]. Few reported studies have used computed tomography angiography (CTA). CTA is an accessible, noninvasive, and relatively easy method of investigation and supports powerful postprocessing; owing to these advantages, CTA has taken on an important role in anatomical research [6].

Therefore, we performed a study of PcomA aneurysms and their adjacent arteries using head CTA. Importantly, the data of the present study were derived from Han Chinese subjects, a population that has rarely been reported in such cases.

## 2. Materials and Methods

Between March 2020 and March 2022, a CTA study was performed on Han Chinese patients at our institution who had ruptured PcomA aneurysms. The ethics committee of our hospital approved this study (No.2022-KS-008). The original CTA data were further processed on a GE Workstation (version 4.7) (GE Healthcare; Cytiva).

### 2.1. Inclusion and Exclusion Criteria

(a) The patient had a subarachnoid hemorrhage (SAH) and underwent CTA examination. (b) On CTA, a unilateral ruptured PcomA aneurysm was diagnosed; on CT, the PcomA aneurysm was observed in the focus of the SAH. (c) Intracranial vessels after contrast agent filling were shown clearly, without occlusions or arteriovenous shunts that could affect the measurement of the parameters. (d) If the PcomA was associated with other aneurysms, they were in locations distal to the PcomA, such as ipsilateral or contralateral middle cerebral artery (MCA), anterior communicating artery, or posterior circulation. (e) The patient did not have other aneurysms near the PcomA, e.g., on ophthalmic artery segment, anterior choroidal artery, or ICA termination that could affect the measurement of the parameters

### 2.2. Grouping

The ICAs ipsilateral to the PcomA aneurysms were considered the aneurysm group, and the contralateral lesion-free ICAs were considered the control group. In addition, some parameters of normal ICA were measured.

### 2.3. Scoring System

Each patient's state was graded on the Hunt and Hess (HH) scale, as follows: Grade I: asymptomatic or minimal headache and slight nuchal rigidity; Grade II: moderate to severe headache, nuchal rigidity, no neurological deficits other than cranial nerve palsy; Grade III: drowsiness, confusion, or mild focal deficits; Grade IV: stupor, moderate to severe hemiparesis, and possibly early decerebrate rigidity and disturbance of vital functions; and Grade V: deep coma, decerebrate rigidity, and a moribund appearance [7].

**Figure 1:**
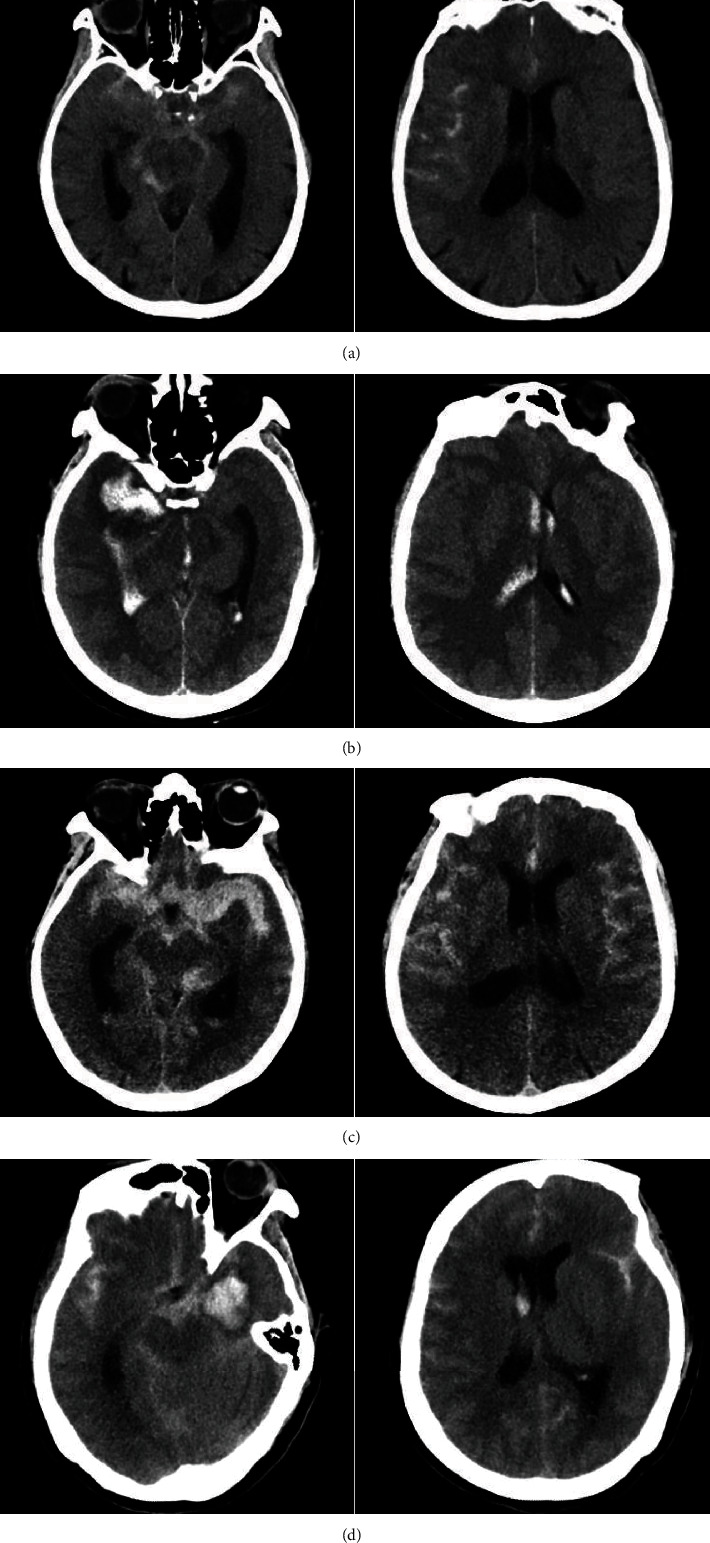
The mFS, a CT-based rating scale is as follows; (a) Grade 1 consists of thin, minimal, or diffuse SAH without IVH; (b) Grade 2 consists of minimal or thin SAH with IVH; (c) Grade 3 is defined by a thick cisternal clot without IVH; (d) Grade 4 is defined by a cisternal clot with IVH. Abbreviations: CT: computed tomography; IVH: intraventricular hemorrhage; mFS: modified Fisher scale; SAH: subarachnoid hemorrhage.

The extent of initial intracranial hemorrhage on CT was recorded using the modified Fisher scale (mFS), whose five levels are defined as follows: Grade 0, no SAH or intraventricular hemorrhage (IVH); Grade 1, focal or diffuse, thin SAH, and no IVH; Grade 2, focal or diffuse, thin SAH, and with IVH; Grade 3, focal or diffuse, thick SAH, and no IVH; and Grade 4, focal or diffuse, thick SAH, and with IVH (Figure 1) [8, 9].

### 2.4. Software and Tools Used for Postprocessing

The raw CTA data were postprocessed using a GE Workstation. The raw CTA data were initially reconstructed using volume rendering procedure. Structures that interfered with the measurement were removed using the cutting tool. The vessel diameter was obtained using the distance-measuring tool. The curved length of a vessel was measured using the two-click AVA tool. The angle between the vessels was measured by the degree tool. Each parameter was measured 3 times, and the average value was used for analysis.

### 2.5. Measured Parameters

**Figure 2:**
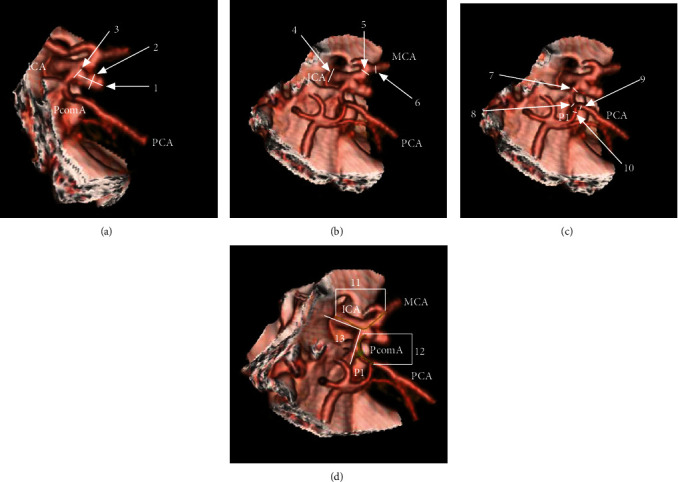
CTA-based measurement parameters are as follows: (a) No. 1 is the height of the aneurysm, No. 2 is the width of the aneurysm, and No. 3 is the width of the aneurysmal neck; (b) No. 4 is the diameter of the intradural ICA at its origin, No. 5 is the diameter of the ICA at its termination, and No. 6 is the diameter of the MCA at its origin; (c) No. 7 is the diameter of the PcomA at its origin, No. 8 is the diameter of the PcomA at its termination, No. 9 is the diameter of P2 at its origin, and No. 10 is the diameter of P1 at its termination; (d) No. 11 is the length of the intradural ICA, No. 12 is the length of the PcomA, and No. 13 is the angle between the ICA and the PcomA. Abbreviations: CTA: computed tomography angiography; ICA: internal carotid artery; MCA: middle cerebral artery; PCA: posterior cerebral artery; PcomA: posterior communicating artery; P1: first segment of PCA.

The height, width, and neck width of the aneurysm were measured (Figure 2(a)). Additionally, diameters of the ICA, PcomA, and posterior cerebral artery (PCA) were measured in different locations (Figures 2(b) and 2(c)). The lengths of intracranial intradural ICA and PcomA and the angle between the proximal ICA and PcomA were measured as well (Figure 2(d)).

### 2.6. Aneurysm Projection and PcomA Development

**Figure 3:**
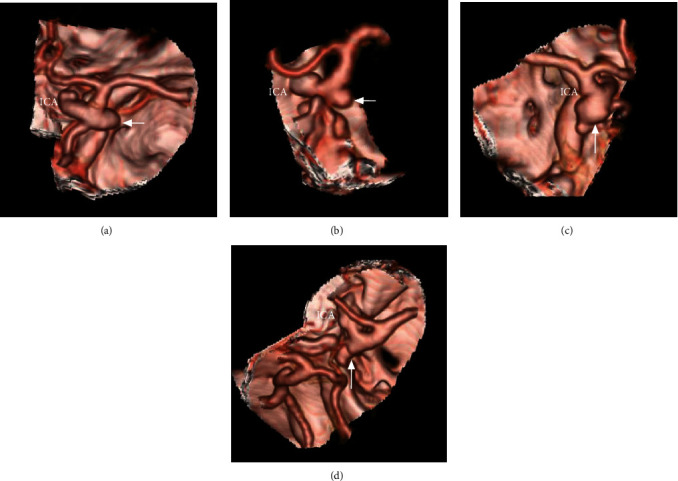
Aneurysm projection in CTA is as folows; (a–b) CTA showing that the aneurysm projected laterally (arrows). (c–d); CTA showing that the aneurysm projected posteriorly (arrows). Abbreviations: CTA: computed tomography angiography; ICA: internal carotid artery.

**Figure 4:**
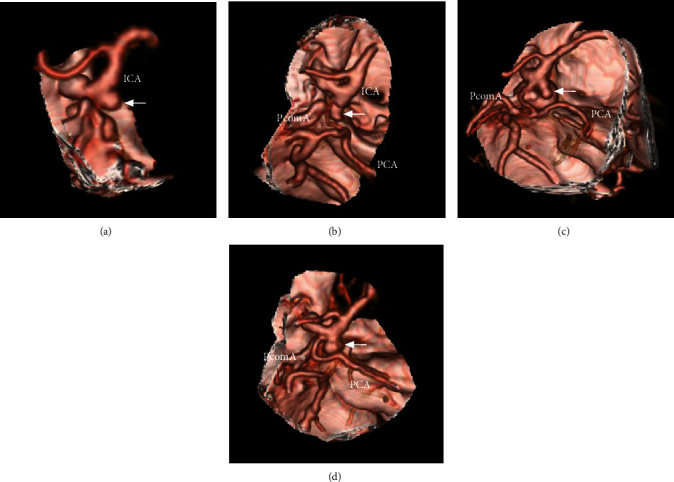
PcomA development as shown by CTA is as follows: (a) Grade I is defined by the absence of the PcomA; the arrow indicates the aneurysm. (b) Grade II is defined by the presence of a thin PcomA (asterisk); the arrow indicates the aneurysm. (c–d) Grade III is defined by the presence of a thick fetal-type PcomA (asterisk); the arrow indicates the aneurysm. Abbreviations: CTA: computed tomography angiography; ICA: internal carotid artery; PCA: posterior cerebral artery; PcomA: posterior communicating artery.

The direction in which the aneurysm projected was classified as either posterior or lateral (Figure 3). The development of the PcomA was divided into three grades: Grade I was defined by the absence of the PcomA; Grade II was defined by the presence of a thin PcomA; and Grade III was defined by the presence of a thick fetal-type PcomA (Figure 4).

### 2.7. Statistical Analysis

Statistical assessments were performed using GraphPad Prism (8.02 (LLC, San Diego, CA, USA)). Continuous variables are expressed as the mean ± standard deviation. A paired *t* test was used for the comparison of two continuous variables. Ordinary one-way ANOVA was used for the comparison of multiple continuous variables. A chi-squared test or Fisher's exact test was used to analyze count data. A *P* < 0.05 was considered to indicate a statistically significant difference.

## 3. Results

### 3.1. General Information

Sixty-three patients who met the inclusion criteria were selected for further investigation. The average age was 62.1 ± 11.0 years (range, 29-82 years), and the ratio of males to females was 0.8 : 1 (28/35). The distribution of HH grades was as follows: Grade I was observed in 36 (57.1%, 36/63) patients, Grade II in 10 (15.9%, 10/63) patients, and Grade III in 17 (27%, 17/63) patients. Regarding mFS scores, Grade 0 was observed in 5 (7.9%, 5/63) patient, Grade I in 24 (38.1%, 24/63) patients, Grade II in 10 (15.9%, 10/63) patients, Grade III in 9 (14.3%, 9/63) patients, and Grade IV in 15 (23.8%, 15/63) patients.

### 3.2. Measured Parameters

PcomA aneurysms were located on the left in 52.4% of patients. The direction of projection was posterior in 68.3% of PcomA aneurysms and lateral in the other 31.7%. In 12.7% of patients with PcomA aneurysms, there were associated distal aneurysms as well. The mean aneurysmal height was 5.2 ± 2.3 mm, and the mean width was 4.7 ± 2.2 mm. The mean aneurysmal neck width was 4.5 ± 1.9 mm. The average size of a ruptured PcomA aneurysm was approximately 5 mm. More information is provided in Table 1.

In the aneurysm group, the intradural ICA diameter was 4.3 ± 0.9 mm, the diameter of the ICA at its termination was 3.6 ± 0.7 mm, the diameter of the MCA at its origin was 2.7 ± 0.5 mm, the diameter of the PcomA at its origin was 2.4 ± 0.8 mm, the intradural ICA length was 19.6 ± 4.5 mm, the PcomA length was 13.7 ± 4.9 mm, and the angle between ICA and PcomA was 68.1 ± 33.3°mm. More information is shown in Table 2. The distribution of PcomA development is shown in Table 3. Grade III fetal-type PcomA was observed in 52.4% of the aneurysm group and 46% of the control group. In addition, the intradural ICA diameter, the diameter of the ICA at its termination, and the intradural ICA length in normal intradural ICAs were provided in Table 4.

### 3.3. Statistical Results

A paired *t* test showed that the difference between the aneurysmal height and width was significant, with the height being greater than the width (*P* < 0.05), which indicates that the typical aneurysm was not spherical (Table 1). Furthermore, paired *t* tests showed that the aneurysm group and the control group significantly differed in intradural ICA diameter, ICA diameter at the termination, intradural ICA length, and ICA-PcomA angle (*P* < 0.05); these differences indicated that, when a PcomA aneurysm was present, the ICA on the affected side became thicker and longer than the contralateral ICA, and the aneurysm reduced the angle between the ICA and the PcomA (Table 2). Additionally, a chi-square test showed no difference in PcomA development between the aneurysm and control groups (*P* > 0.05) (Table 3).

Intradural ICA diameter at origin, ICA diameter at termination, and intradural ICA length among normal bilateral ICAs and lesion-free ICA in patients with PcomA aneurysm were compared using ordinary one-way ANOVA; the *P* value was >0.05, and no differences were found (Table 4).

## 4. Discussion

The parameters of a PcomA aneurysm and its adjacent arteries are very important [10, 11]. Previous studies have used catheter-based angiography procedures and anatomical specimens to study the PcomA and adjacent arteries [3, 4, 12]. CTA plays an important role in anatomical research owing to its speed and accessibility [13, 14]. Therefore, in our study, PcomA aneurysms and nearby vessels were studied using CTA. Importantly, this study analyzed data from Han Chinese people, who have not been extensively studied.

Based on International Subarachnoid Aneurysm Trial (ISAT), aneurysms are categorized as small (<7 mm), medium (7-12 mm), large (>12-25 mm), or giant (>25 mm) [15]. Among small aneurysms, PcomA aneurysms were associated with a relatively higher risk of rupture [16]. In Forget et al.'s report, the proportion of small lesions among ruptured PcomA aneurysms was particularly high, with 87.5% of these aneurysms measuring less than 10 mm in diameter and 40% measuring less than 5 mm in diameter [17]. In our study, the overall average size of ruptured PcomA aneurysms was approximately 5 mm (less than the cutoff value of 7 mm), also, demonstrating that small aneurysms were susceptible to rupture in the Han Chinese population.

According to the current standards, wide-necked intracranial aneurysms are defined by a neck width ≥4 mm or a dome-to-neck ratio<2 [18]. In a previous report, PcomA aneurysms were frequently found to have wide necks [19]. In our study, the mean aneurysmal neck width was 4.5 mm, which indicated that nearly all of ruptured PcomA aneurysms were wide-necked. The direction in which an aneurysm projects is an important factor to consider, especially in endovascular treatment [20]. For catheterization, PcomA aneurysms that project in the posterior direction need only a microcatheter with a simple C shape. In contrast, those that project laterally need a microcatheter with a complex C shape, where left or right rotation is added to the basic curve. Therefore, microcatheter navigation is easier if the aneurysm projects posteriorly than if it projects laterally. Fortunately, posterior projection is common. [21] In this study, 68.3% of ruptured PcomA aneurysms projected in the posterior direction, which made catheterization easy.

PcomA variation was common, ranging from absence to the fetal type (Figure 4). A fetal-type PcomA or PCA is defined as a PcomA that has the same caliber as the P2 segment of the PCA and is associated with an atrophic P1 segment. In a previous report, the rate of fetal-type PcomA was estimated to occur in 4.4-40% of individuals [22, 23]. In our study, fetal-type PcomA was 52.4% in aneurysm group and 46% in control group without the difference between these two groups. The incidence of fetal-type PcomA in our study was higher than that of 4.4-40% in the above reports. The reason may be that PcomA aneurysms are often associated with fetal-type PcomA, because a fetal-type PcomA can lead to increased PcomA pressure, resulting in development and rupture of PcomA aneurysms; therefore, in individuals with PcomA aneurysms, the fetal-type PcomA was popular [24, 25]. In addition, in our study, due to no difference in PcomA development between the aneurysm and control groups, the fetal-type PcomA structure tended to be bilateral.

The angle between the proximal ICA and the normal, lesion-free PcomA has rarely been measured in previous research; in our study, the mean angle was 87.9 ± 27.2°mm. Due to the space-occupying effect of the aneurysm, the PcomA may be pushed, changing the angle [26]. In a study by Yuan et al., the mean angle in a PcomA aneurysm side was 66.3°mm [27]. Similarly, in our study, the mean angle in the aneurysm group was 68.1 ± 33.3°mm. This angle was significantly less than that of the control group, which indicated that the PcomA may be pushed by the aneurysm toward the proximal ICA, narrowing the space between them.

It is feasible to compare the diameters and lengths of the intracranial ICA on the sides with and without the aneurysm to determine whether the lesion has changed these parameters [5]. In our study, statistical analysis revealed that intradural ICA origin diameter, ICA termination diameter, and intradural ICA length were significantly different between the aneurysm and control groups, other diameters and lengths of the vessels had no differences. Were the thickening and lengthening of intradural ICA in aneurysm group caused as a result of the aneurysm? Or was the intradural ICA always thicker and longer than that of the control side, but just happened to have an aneurysm associated with it?

In our study, we compared the intradural ICA diameter at origin, ICA diameter at termination, and intradural ICA length among normal bilateral ICAs and lesion-free ICA in patients with PcomA aneurysms and found no differences (Table 4). Therefore, it could be that a larger and longer intradural ICA in the aneurysm group might imply more hemodynamic shear stress.

This study also provides the above important reference data on the Han Chinese population, which may help surgeons choose the appropriate sizes of endovascular treatment products, as follows: when a PcomA aneurysm was found, the mean intradural ICA diameter was 4.3 mm, the mean diameter of the ICA at its termination was 3.6 mm, and the mean intradural ICA length was 19.6 mm (Table 2).

## 5. Conclusions

This CTA study showed that the typical ruptured PcomA aneurysm was a wide-necked, nonspherical lesion approximately 5 mm in size. A fetal-type PcomA was found in cases with a PcomA aneurysm. In the presence of a ruptured PcomA aneurysm, the ICA on the affected side became thicker and longer than the contralateral control vessel, and the aneurysm significantly changed the angle between the ICA and the PcomA. Additionally, this study obtained important reference data for the Han Chinese population, which could be consequential for clinical practice.

## Figures and Tables

**Table 1:** Aneurysm data.

AN side	Left	33 (52.4%, 33/63)	
	Right	30 (47.6%, 30/63)	

Direction of AN projection	Posterior	43 (68.3%, 43/63)	
	Lateral	20 (31.7%, 20/63)	

Associated ANs	No	55 (87.3%, 55/63)	
	Yes	8 (12.7%, 8/63)	

AN size	Height	5.2 ± 2.3 mm (range, 1.1-13.6 mm)	*P* = 0.0071
	Width	4.7 ± 2.2 mm (range, 1.0-13.7 mm)	

AN neck width	4.5 ± 1.9 mm (range, 1.7-12.4 mm)		

In this table, a paired *t* test was used to evaluate the difference between the height and width of the aneurysm. Abbreviations: AN: aneurysm.

**Table 2:** Statistical analysis between the aneurysm and control groups.

Parameter	Groups	Range (mm)	Mean (mm)	*P* *value*
Intradural ICA diameter at origin	AN (63)	2-6.5	4.3 ± 0.9	0.0293
	Control (63)	2.5-5.7	4.1 ± 0.8	

ICA diameter at termination	AN (63)	2.2-6.2	3.6 ± 0.7	0.0024
	Control (63)	2.0-4.7	3.3 ± 0.6	

MCA diameter at origin	AN (63)	1.0-3.9	2.7 ± 0.5	0.4441
	Control (63)	1.6-3.9	2.8 ± 0.5	

PcomA diameter at origin	AN (41)	0.3-4.4	2.4 ± 0.8	0.2815
	Control (40)	0.5-4.2	2.6 ± 0.8	

PcomA diameter at termination	AN (41)	0.4-3.2	1.8 ± 0.6	0.3739
	Control (40)	0.3-2.6	1.7 ± 0.7	

P2 diameter at origin	AN (41)	0.4-3.2	2.1 ± 0.6	0.9862
	Control (40)	0.4-3.1	2.1 ± 0.5	

P1 diameter at termination	AN (41)	0-2.3	0.9 ± 0.8	0.6303
	Control (40)	0-2.6	1.0 ± 0.9	

Intradural ICA length	AN (63)	11-34.7	19.6 ± 4.5	0.0089
	Control (63)	11.5-28.7	18.2 ± 3.7	

PcomA length	AN (41)	1.7-26.6	13.7 ± 4.9	0.2311
	Control (40)	9.3-19.4	14.8 ± 2.7	

Angle between ICA and PcomA	AN (41)	9.8-146.5	68.1 ± 33.3	0.0045
	Control (40)	27.6-172.5	87.9 ± 27.2	

In this table, a paired *t* test was used for the following parameters: intradural ICA diameter at the origin, ICA diameter at the termination, MCA diameter at the origin and intradural ICA length. An unpaired *t* test was used for other parameters. Abbreviations: AN: aneurysm; ICA: internal carotid artery; MCA: middle cerebral artery; P1: first segment of the posterior cerebral artery; P2: second segment of the posterior cerebral artery; PcomA: posterior communicating artery.

**Table 3:** PcomA development.

Group	Grade I	Grade II	Grade III	P value
AN (63)	22	8	33	0.6859
Control (63)	23	11	29	

In the table, a chi-squared test was used. Abbreviations: AN: aneurysm; PcomA: posterior communicating artery.

**Table 4:** Statistical analysis among the left and right normal ICAs and the control group.

Parameter	Groups	Range (mm)	Mean (mm)	*P* value
Intradural ICA diameter at origin	Normal L (30)	2.6-5.2	4.2 ± 0.6	0.3284
	Normal R (30)	2.6-5.6	4.3 ± 0.7	
	Control group (63)	2.5-5.7	4.1 ± 0.8	

ICA diameter at termination	Normal L (30)	1.7-4.0	3.3 ± 0.5	0.4823
	Normal R (30)	2.1-4.3	3.3 ± 0.6	
	Control (63)	2.0-4.7	3.3 ± 0.6	

Intradural ICA length	Normal L (30)	11.7-25.7	17.1 ± 3.3	0.2674
	Normal R (30)	11.9-29.9	18.6 ± 4.1	
	Control (63)	11.5-28.7	18.2 ± 3.7	

In this table, ordinary one-way ANOVA was used. Abbreviations: ICA: internal carotid artery; L: left; R: right.

## Data Availability

All raw data will be available on the request.
